# Case Report: Combination Therapy With PD-1 Blockade for Acute Myeloid Leukemia After Allogeneic Hematopoietic Stem Cell Transplantation Resulted in Fatal GVHD

**DOI:** 10.3389/fimmu.2021.639217

**Published:** 2021-04-01

**Authors:** Sun Yao, Chen Jianlin, Qiao Zhuoqing, Li Yuhang, Hu Jiangwei, Hu Guoliang, Ning Hongmei, Zhang Bin, Hu Liangding

**Affiliations:** ^1^Department of Hematology, The Fifth Medical Center of Chinese People's Liberation Army General Hospital, Beijing, China; ^2^Institute of Hematology, The Fifth Medical Center of Chinese People's Liberation Army General Hospital, Beijing, China; ^3^Beijing Key Laboratory of Hematopoietic Stem Cell Therapy and Transformation Research, Beijing, China

**Keywords:** acute myeloid leukemia, post-transplantation relapse, GvHD, immune checkpoint blockade, hypomethylating agents

## Abstract

**Background:** Azacitidine is commonly used in the treatment of relapsed acute myeloid leukemia (AML) and myelodysplastic syndrome (MDS) after allogeneic hematopoietic stem cell transplantation (allo-HSCT), but the effectiveness of this monotherapy is still very low. A possible mechanism of resistance to hypomethylating agents (HMAs) is the upregulation of the expression of inhibitory checkpoint receptors and their ligands, making the combination of HMAs and immune checkpoint blockade therapy a rational approach. Although the safety of anti-programmed cell death protein (PD)-1 antibodies for patients with post-allo-HSCT remains a complicated issue, the preliminary clinical result of combining azacitidine with anti-PD-1 antibodies is encouraging; however, the safety and efficacy of this approach need further investigation.

**Case Presentation:** We reported a case of treated secondary (ts)-AML in a patient who received tislelizumab (an anti-PD-1 antibody) in combination with azacitidine. The patient relapsed after allo-HSCT and was previously exposed to HMAs-based therapy. The patient received tislelizumab for compassionate use. After the combination treatment, the patient achieved complete remission with incomplete hematologic recovery, negative minimal residual disease (MRD) by flow cytometry (FCM), and negative Wilms' tumor protein 1 (WT1). However, the patient successively developed serious immune-related adverse events (irAEs) and graft vs. host disease (GVHD) and eventually died from complications of GVHD.

**Conclusion:** To our knowledge, this is the first case to report the combined use of tislelizumab and azacitidine to treat relapsed AML posttransplantation. This report highlights the safety concerns of using an anti-PD-1 antibody in combination with azacitidine after allo-HSCT, especially the risk of GVHD, and provides a basis for future studies.

## Introduction

Although allogeneic hematopoietic stem cell transplantation (allo-HSCT) is a potentially curative therapy for patients with high-risk acute myeloid leukemia (AML) and myelodysplastic syndrome (MDS), the relapse of the disease remains the major cause of treatment failure in these patients and carries a dismal prognosis (1–4). Hypomethylating agents (HMAs), such as azacitidine and decitabine, are the most common, non-targeted pharmacologic agents used to treat and prevent the relapse in posttransplantation AML and MDS in recent times. However, a single-agent HMA therapy in relapsed/refractory (r/r) HMAs-naïve AML has only achieved a low response rate (5–8). Previous studies have shown that, while HMAs promote antitumor immune signaling (9), they concurrently dampen antitumor immunity by increasing the expression of programmed cell death protein (PD)-1 and programmed death-ligand (PD-L)1 in solid tumors (10) and MDS/AML (11). This could be a possible mechanism of resistance to HMAs (8). For patients with relapsed AML after human leukocyte antigen (HLA) matching and incompatible transplantation without HLA loss, the mechanism of recurrence after the transplantation is mainly by the downregulation of HLA class two molecules (30–40%) and the upregulation of immune checkpoints (~20%) at the epigenetic level, which can be treated by HMAs and immune checkpoint blockade (ICB) therapy, respectively (12). Thus, for posttransplantation AML, the combination therapy of azacitidine with anti-PD-1 antibody may be a better approach in comparison to monotherapy. In fact, single-agent anti-PD-1 antibodies exhibit only minimal activity in patients with relapsed AML and high-risk MDS (13–15). ICB therapy after allo-HSCT has been reported to cause severe graft vs. host disease (GVHD) in both preclinical (16–18) and clinical studies (15, 19–22). However, the combination therapy of azacitidine and nivolumab (an anti-PD-1 antibody) showed an encouraging response with no GVHD and moderate immune-related adverse events (irAEs) with respect to the relapse of AML/MDS (prior allo-HSCT-19%) in a clinical trial (8). Given these promising preliminary clinical results, the safety and efficacy of combining azacitidine and anti-PD-1 antibodies in post-allo-HSCT patients should be urgently investigated further. Tislelizumab® (BeiGene, China), an antihuman PD-1 monoclonal IgG4 antibody, has been approved in China for patients with r/r classical Hodgkin lymphoma (HL) after at least a second-line chemotherapy (23). In the present study, we report a case of compassionate use of tislelizumab combined with azacitidine to treat a patient with relapsed AML after allo-HSCT. The report highlights the importance of the prudent use of an anti-PD-1 antibody in patients who are undergoing HSCT.

## Case Presentation

**Figure 1:**
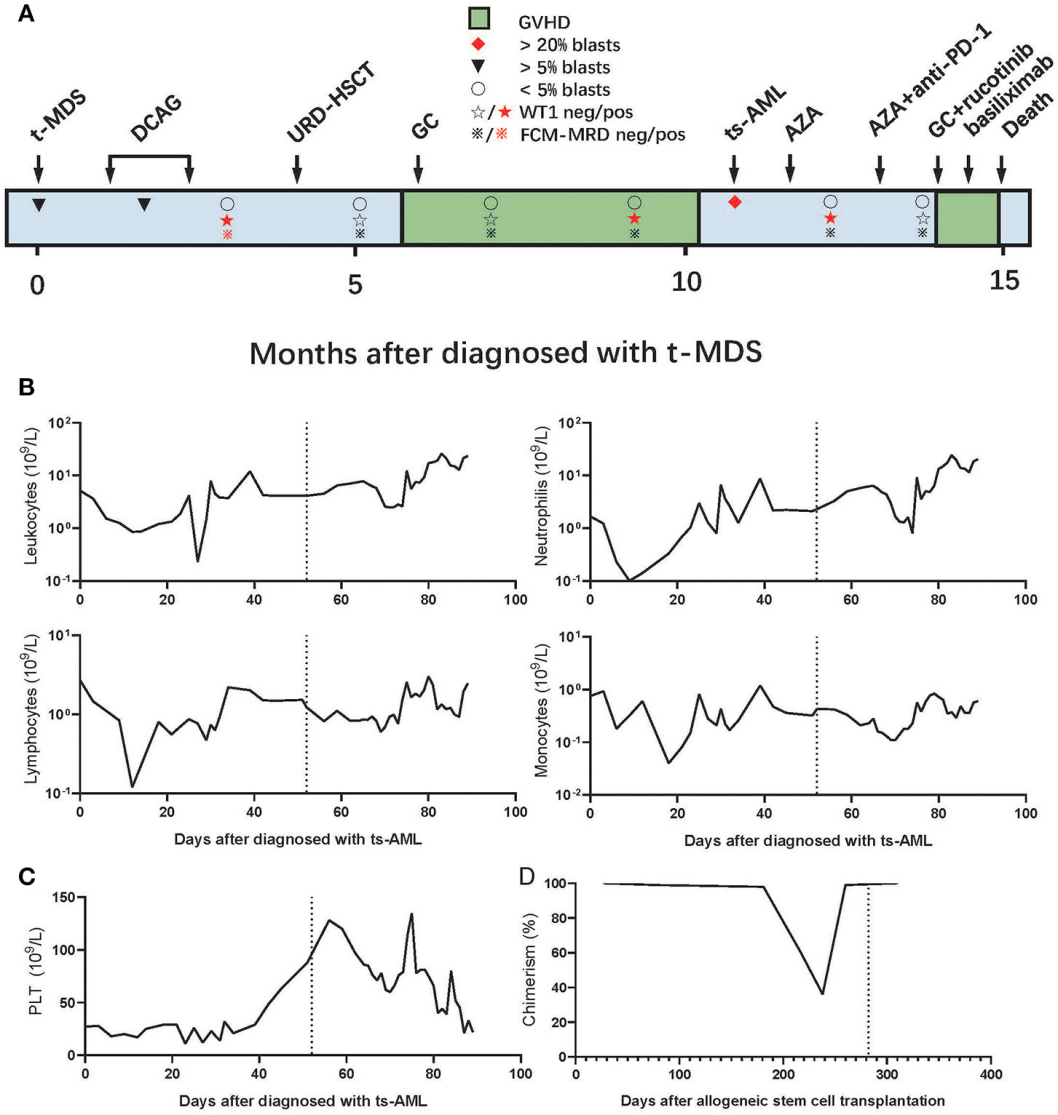
**(A)** Clinical course of the patient. **(B,C)** Numbers of **(B)** leukocytes, lymphocytes, neutrophils, monocytes, and **(C)** platelets in the peripheral blood after the AML diagnosis. **(D)** Donor cell chimerism in the bone marrow (BM) following allo-HSCT. In **(B–D)**, the dotted vertical line indicates the timing of tislelizumab administration. GC, glucocorticoid; AZA, azacytidine; t-MDS, therapy-related myelodysplastic syndrome; ts-AML, treated secondary-acute myeloid leukemia. URD-HSCT, unrelated donor hematopoietic stem cell transplantation.

A 56-year-old man was diagnosed with follicular lymphoma [FL; grade IIIA, stage IVA, Follicular Lymphoma International Prognostic Index (FLIPI) stage: high risk] 18 years ago. The patient was cured by four sequential cycles of fludarabine, cyclophosphamide, rituximab (FCR) chemotherapy; four cycles of rituximab, cyclophosphamide, hydroxyldaunorubicin, oncovin, and prednisone (R-CHOP) chemotherapy; and local lymph node radiotherapy. Unfortunately, the patient was diagnosed with therapy-related MDS (t-MDS) in February 2019 according to the WHO classification (Figure 1A). The baseline characteristics of the patient diagnosed with t-MDS are presented in the Supplementary Material.

The patient received induction chemotherapy with a decitabine, cytarabine, aclacinomycin, and recombinant human granulocyte colony-stimulating factor (G-CSF) (DCAG) scheme in March 2019 and achieved a partial response (PR). Then, the patient received another cycle of consolidation chemotherapy with DCAG and achieved a complete response (CR); at this stage, the patient was positive for minimal residual disease (MRD), confirmed through flow cytometry (FCM). The patient underwent allo-HSCT from a HLA-mismatched unrelated donor (8/10), after preconditioning with decitabine, fludarabine, and busulfan, followed by cyclosporine A, mycophenolate mofetil, basiliximab (a monoclonal anti-CD25 antibody), and short-term methotrexate for prophylaxis of GVHD. The patient achieved CR with MRD negativity (CR_MRD_-) 1 month after allo-HSCT and developed extensive skin chronic GVHD (cGVHD) and bronchiolitis obliterans with organizing pneumonia (BOOP) 6 months after allo-HSCT but improved after glucocorticoids and antifungal therapy. During the treatment for BOOP, the patient remained CR_MRD_- but was positive for Wilms' tumor protein 1 (WT1+). In January 2020, the disease progressed to AML, and the evaluation of bone marrow (BM) showed that 34.5% of blasts, 36.14% of donor chimeric,; 28.8% of FCM–MRD; and 7.57% of WT1. The patient was diagnosed with treated secondary (ts)-AML, arising from an antecedent hematologic disorder that was previously treated with chemotherapy or immunomodulatory therapy, an entity known to have an extremely dismal prognosis (24–26). The gene mutation test from a BM sample showed casitas B-lineage lymphoma (CBL) of 5.92% and Kirsten rat sarcoma (KRAS) of 6.3%. The immunosuppressor was immediately withdrawn. We performed the HLA-loss test, but no HLA gene loss was detected.

The patient was counseled on the risks and benefits of azacitidine in combination with tislelizumab. Although the patient did not have any signs or symptoms of GVHD at the time of relapse, we decided to administer anti-PD-1 after one course of azacitidine to ensure the use of tislelizumab for at least 4 weeks after the withdrawal of immunosuppressive agents according to a previous study (15). Thus, the patient received azacitidine monotherapy and achieved 0.611% of CRi_MRD_- and WT1 1 month later. The patient subsequently developed herpes zoster infection, but the condition of the patient improved with antiviral therapy. The patient also developed a drug-induced liver injury, but the condition of the patient improved after the drugs causing liver injury were discontinued, namely estazolam and zopiclone, which had been prescribed for insomnia. In March 2020, the patient received 100 mg of azacitidine on days 1–7 subcutaneously and 200 mg of tislelizumab on day 1 intravenously. About 20 days later, the patient remained CRi_MRD_- and was WT1 negative (WT1–) (0.11%, the cutoff value of WT1/ABL in our laboratory is 0.5%). The patient successively developed hypoadrenocorticism, infectious diarrhea, fever, and shock. Although the symptoms of the patient were relieved with symptomatic and antimicrobial treatment, diarrhea continued to worsen. No definite infection was found after repeated etiological examinations, and multiple antibiotic treatments proved to be ineffective.

The patient refused to undergo a colonoscopy and further biopsies, so the diagnosis of G3 gut acute GVHD (aGVHD) was mainly based on history and clinical manifestation. Prednisone, 2 mg/kg/day, combined with ruxolitinib, 10 mg (bid), was prescribed. The patient continued to have diarrhea even after 5 days. Prednisone was tapered and basiliximab was started. The patient subsequently developed delirious behavior, involuntary tremors, decreased muscle strength, and dystonia. A diagnosis of autoimmune-related encephalopathy was hypothesized after consultation with a neurologist, based on history, clinical manifestations, and imaging. CT showed multiple spots and patches of low-density lesions around bilateral lateral ventricles, and MRI showed scattered spots and patchy lesions near both frontal lobes and lateral ventricles that showed equal or long signal on T1 images, a long signal on T2 images, and a high signal on T2WI fluid-attenuated inversion recovery (FLAIR). Gamma globulins were administered, but the nervous system symptoms were not relieved. Gut aGVHD was resistant to steroid and second-line treatment, and the patient subsequently developed hematochezia, enteric infections, septic shock, and metabolic acidosis secondary to gut GVHD and died 6 days later (Figures 1B–D).

## Discussion and Literature Review

The patient with MDS mentioned in the study was previously exposed to HMA therapy, which rapidly progressed to ts-AML after allo-HSCT. At the time of relapse, neither HLA loss nor active GVHD was present. First, the patient received azacitidine monotherapy and achieved CRi_MRD_- but was WT1+. Subsequently, the patient received a combination of azacitidine and tislelizumab and remained CRi_MRD_- and became WT1-. Unfortunately, the patient developed serious irAEs, including hypoadrenocorticism, autoimmune-related encephalopathy, and fatal gut GVHD. We have summarized the safety and efficacy of using checkpoint inhibitors in post-allo-HSCT myeloid malignancies in Table 1. Clinical studies showed that the CTLA-4 blockade induces lower GVHD as compared to anti-PD-1 (14% vs. 39%) (15, 28). Furthermore, CTLA-4 inhibitors as single agents demonstrated activity in patients with high-risk MDS after the therapy of HMAs and relapsed AML post-allo-HSCT, while anti-PD-1 antibodies showed limited efficacy (28, 34). Currently, clinical trials of the combination of HMAs with ICB therapy are ongoing (8, 34). Table 2 shows a summary of autoimmune complications of the published clinical trials using checkpoint inhibitors in post-allo-HSCT hematologic malignancies other than myeloid malignancies. On the whole, the incidence of autoimmune diseases, including GVHD, after ICB monotherapy is high: 21–39% in AML/MDS (15, 28) and 30%−55% in other hematological malignancies (20, 36).

**Table 1:** The safety and efficacy of the published clinical trials of immune checkpoint blockade in post-allo-HSCT myeloid malignancies.

**References**	**Immune checkpoint inhibitors/ pathway**	**HMAs**	**Study design**	**Trial regimen**	**Study population (N)**	**Efficacy**	**Safety**
**ICB THERAPY ONLY**							
Bashey et al. (27)	Ipilimumab/CTLA-4	-	Phase 1	Single arm in relapsed Malignancies after allo-HSCT	Total (29) AML (2) CML (2)	No response in the four patients	In all myeloid malignancies after allo-HSCT, no patient developed DLT and GVHD. One patient with AML developed G3 polyarthropathy with nodules clinically consistent with rheumatoid arthritis.
Davids et al. (28)	Ipilimumab/CTLA-4	-	phase 1/1b	Single arm in relapsed HMs After allo-HSCT	Total (28) AML (12) Relapse with extramedullary disease (4) MDS (2) MPN (1)	In myeloid malignancies, four patients with extramedullary and one patient with MDS/AML achieved CR.	In all patients, 6 (21%) developed irAEs including 1 death, 4 (14%) developed GVHD. All of GVHD resolved with glucocorticoids. Other sAEs: acute kidney injury, corneal ulcer, thrombocytopenia, neutropenia, anemia, and pleural effusion. ^*^
Holderried et al. (22)	Nivolumab/PD-1 Ipilimumab/CTLA-4	-	Retrospective study	Disease recurrence after allo-HSCT other than HL	Total (21) AML/MDS (12)	One patient with AML received Niv + DLI survived > 2 years after Niv with ongoing CR. One AML received Niv survived > 2 years after Niv with PD.	2/12 patients with AML/MDS developed GVHD. One received Niv, the other one received Niv + Ipi.
Wong et al. (29)	Nivolumab/PD-1	-	Phase 2a	Single arm in relapsed or persistent HMs after allo-HSCT	Total (6) AML (2)	One patient with AML achieved transient blast reduction but progressed subsequently.	2/6 patents with HMs developed G3 aGVHD 2 weeks after first dose of Niv. ^*^
Davids et al. (15)	Nivolumab/PD-1	-	Phase 1	Single arm in relapsed HMs after allo-HSCT	Total (28) AML (10) MDS (7) CMML (1)	less activity in patients with myeloid malignancies (ORR 21%).	11 HMs pts (39%) developed new or worsening a/c-GVHD (two acute, eight chronic, and one both). Additional sAEs: pneumonitis, transaminitis, respiratory syncytial virus pneumonia, rash, orthostatic hypotension, and lipase elevation. ^*^
Schoch et al. (30)	Nivolumab/PD-1 Pembrolizumab/PD-1 Ipilimumab/CTLA-4	-	Retrospective study	Relapsed cancers afterallo-HSCT.	Total (9) AML (1) MDS (1)	^*^	In all the 9 patients (including two with solid tumors), one developed G2 cutaneous aGVHD when DLI was given for relapsed disease after ipilimumab. ^*^
Liao et al. (31)	Pembrolizumab/PD-1	-		Single arm in relapsed AML after allo-HSCT	AML (8)	No response	Can induce early and severe irAEs.
Wang et al. (32)	Nivolumab/PD-1	-	Cases report	Maintenance therapy after allo-HSCT in myeloid malignancies	AML (3) t-MDS (1)	-	All the 4 patients rapidly developed irAEs, 2 of them ≥G3.
Albring et al. (33)	Nivolumab/PD-1	-	Cases report	Monotherapy in relapsed AML after allo-HSCT	AML (3)	1 CR, 1 SD, 1 NR	Pancytopenia and skin GVHD in one patient, muscle and joint pain in another. No severe GVHD.
**HMAs+ICB**							
Daver et al. (8)	Nivolumab/PD-1	AZA	Phase 2	single arm in R/R AML	AML (70) Post-allo-HSCT (13) Post-transplantation AML (13)	ORR 33%, CRR 22% in all patients, ORR 58% in HMAs-naïve and 22% in HMAs-pre-treated patients. ORR 13% in post-allo-HSCT r/r AML.	Grade 3–4 irAEs occurred in 8/70 (11%) R/R AML patients. No GVHD was reported.

*R/R, Relapsed/refractory; NR, Not reported; ORR, Overall response rate; OS, Overall survival; CR, Complete remission; CRR, Complete remission rate; PR, Partial response; HMAs, Hypomethylating agents; ICB, Immune checkpoint blockade; allo-HSCT, Allogeneic hematopoietic stem cell transplantation; SD, Stable disease; PD, Progressive disease; GVHD, Graft vs. host disease; DLT, Dose-limiting toxicity; AML, Acute myeloid leukemia; MDS, Myelodysplastic syndrome; CML, Chronic myeloid leukemia; CMML, Chronic myelomonocytic leukemia; HL, Hodgkin lymphoma; DLI, Donor lymphocyte infusion; irAEs, immune-related adverse events; AZA, Azacytidine; ITP, Immune thrombocytopenic purpura; HMs, Hematological malignancies; MPN, Myeloproliterative neoplasms*.

* *Detailed data about the separate disease are unavailable*.

**Table 2:** Autoimmune complications of the published clinical trials using checkpoint inhibitors in post-allo-HSCT hematologic malignancies other than myeloid malignancies.

**References**	**immune checkpoint inhibitors/pathway**	**Other drugs**	**Study design**	**Trial regimen**	**Study population (N)**	**Autoimmune complications**
Bashey et al. (27)	Ipilimumab/CTLA-4	-	Phase 1	Single arm in relapsed malignancies after allo-HSCT	Total (29) HL (14) Myeloma (6) CLL (2) NHL (1)	3 patients developed organ-specific irAEs, including G2 hyperthyroidism, recurrent G4 pneumonitis, and G3 dyspnea.
Davids et al. (28)	Ipilimumab/CTLA-4	-	Phase 1/1b	Single arm in relapsed HMs after allo-HSCT	Total (28) HL (7) NHL (4) MM (1) ALL (1)	In all patients, 6 (21%) developed irAEs including 1 death, 4 (14%) developed GVHD. ^*^
Holderried et al. (22)	Nivolumab/PD-1 Ipilimumab/CTLA-4	-	Retrospective study	Disease recurrence after allo-HSCT other than HL	Total (21) ALL (2) NHL (5) MF (2)	4/9 patients with non-myeloid hematologic malignancies developed GVHD, 1 received Niv, 3 received Niv + DLI.
Wong et al. (29)	Nivolumab/PD-1	-	phase 2a	Single arm in relapsed or persistent HMs after allo-HSCT	Total (6) HL (2) tCLL (1) MCL (1)	2/6 HMs patients developed G3 aGVHD 2. ^*^
Khouri et al. (35)	Ipilimumab/CTLA-4	Lenalidomide	Phase ii	Relapsed lymphomas after allo-HSCT and high-risk patients after autologous HSCT	17 pts (10 allo, 7 auto)	Allogeneic: 1 cGVHD of liver, mouth, 1 G2 hypothyroid; Autologous: 1 G2 dermatitis, 1 G1 hypothyroid.
Schoch et al. (30)	Nivolumab/PD-1 Pembrolizumab/PD-1 Ipilimumab/CTLA-4	-	Retrospective study	relapsed cancers after allo-HSCT.	Total (9) HL (4) Dsmoplastic small round cell tumor (1)	In all the 9 patients (including 2 with solid tumors), 1 developed G2 cutaneous aGVHD when DLI was given for relapsed disease after ipilimumab. ^*^
Davids et al. (15)	Nivolumab/PD-1	-	Phase 1	Single arm in relapsed HMs after allo-HSCT	Total (28) HL (5) NHL (3) CLL (1)	11 HMs patients (39%) developed new or worsening a/c-GVHD (2 acute, 8 chronic, and 1 both). ^*^
Herbaux et al. (36)	Nivolumab/PD-1	-	Retrospective study	HL patients relapsing after allo-HSCT.	r/r HL (20)	30% (6/20) patients developed GVHD, all of them had prior history of aGVHD. 1 developed possibly related G2 hepatic cytolysis.
Haverkos et al. (20)	Nivolumab/PD-1 Pembrolizumab/PD-1	-	Retrospective study	Relapsed lymphomas after allo-HSCT	HL (29) Other lymphomas (2)	55% (17/31) patients developed treatment-emergent GVHD (6 acute, 4 overlap, and 7 chronic). 29% developed ≥G3 a/cGVHD. 26% deaths related to GVHD. Only 2 of these 17 achieved CR to GVHD treatment, and 14/17 required ≥2 systemic therapies. The majority experienced cutaneous and hepatic GVHD.
Angenendt et al. (37), Covut et al. (38), and Shad et al. (39)	Nivolumab/PD-1	-	Cases report	Relapsed HL after allo-HSCT	HL (4)	None
Chan et al. (40) and Villasboas et al. (41)	Pembrolizumab/PD-1	-	Cases report	Relapsed lymphoma after allo-HSCT	HL (2) ALCL (1)	None
Singh et al. (19)	Pembrolizumab/PD-1	-	Case report	Relapsed HL after allo-HSCT	HL (1)	Stage IV skin, stage II gut and stage IV liver leading to an overall grade IV aGvHD.
Kwong et al. (42)	Pembrolizumab/PD-1	-	Cases report	Relapsed or refractory NK/T-cell lymphoma after allo-HSCT	NK/T-cell lymphoma (7)	G2 skin GVHD disease in 1 patient with previous allo-HSCT.
Godfrey et al. (43)	Nivolumab/PD-1	-	Cases report	Relapsed HL after allo-HSCT	HL (3)	G3 polyarthritis in 1 patient, G2 keratoconjunctivits in 2, G1 rash (possibly representing limited-stage chronic GVHD) in 1.
Boekstegers et al. (44)	Pembrolizumab/PD-1	-	Case report	Relapsed ALL after allo-HSCT	ALL (1)	G4 aGVHD of the skin, mucosa, liver, lung, CNS and eyes. A severe lethal inflammatory disease.
El Cheikh et al. (45)	Nivolumab/PD-1	-	Cases report	Relapsed HL after allo-HSCT	HL (2)	G3 aGVHD involving ocular, liver and skin in 1 patient, G3 aGVHD involving skin, GI and liver in the other pt.
Yared et al. (46)	Nivolumab/PD-1	-	Case report	Relapsed HL after allo-HSCT	HL (1)	G2 pneumonitis and hepatitis

*NHL, Non-Hodgkin's lymphoma; ALL, Acute lymphoblastic leukemia; CLL, Chronic lymphoblastic leukemia; ALCL, Anaplastic large cell lymphoma; R/R, Relapsed/refractory; CR, Complete remission; CRR, Complete remission rate; allo-HSCT, Allogeneic hematopoietic stem cell transplantation; GVHD, Graft vs. host disease; HL, Hodgkin lymphoma; DLI, Donor lymphocyte infusion; irAEs, immune-related adverse events; DLT, Dose-limiting toxicity; MF, Marrow failure; MM, Multiple myeloma; tCLL, transformed chronic lymphocytic leukemia; MCL, Mantle cell lymphoma; HMs, Hematological malignancies*.

* *Detailed data about separate disease are unavailable*.

A possible pathogenic mechanism of GVHD in the patient could involve enteric infection that may have damaged gastrointestinal tissue, favoring T-cell activation against self-antigens. The blockage of PD-1/PD-L1 increases the proliferation, activation, Th1 cytokine-production, and metabolic stress of donor T cells, along with increased homing in the GVHD target tissues such as the gut, due to the loss of intestinal epithelial integrity (47). Moreover, the blockage of PD-1/PD-L1 accelerated donor CD8+ T-cell expansion and exacerbated aGVHD (48). It is challenging to distinguish between gut GVHD and GI-irAEs even after biopsies. We diagnosed a gut aGVHD for the following reasons: first, the patient had a history of cGVHD and was more likely to be susceptible to develop GVHD after the treatment of PD-1 as described in the previous studies (36, 49). Meanwhile, the patient remained completely donor chimeric after anti-PD-1 therapy. However, other studies have suggested that prior a/cGVHD has no significant impact on the development of GVHD after ICB therapy (15, 22). Although this issue is controversial and remains to be clarified, it is a possibility that deserves attention. In addition, the cumulative incidence of gastrointestinal aGVHD might be as high as 60% (50), while the incidence of diarrhea was 11–17% after the treatment of anti-PD-1 (51).

Previous studies showed that 0.5 mg/kg of nivolumab monotherapy for every 3 weeks and 100 mg of nivolumab plus azacitidine for every 2 weeks are considered safe (8, 15). In addition, the low affinity of tislelizumab for the Fc receptor and Fc-γ receptor 1 (FcγRI) may contribute to improved anticancer efficacy as compared to other anti-PD-1 antibodies (52), which means that the dose of tislelizumab may need to be further reduced. It is interesting that the patient developed a delayed and steroid-resistant GVHD nearly 4 weeks after anti-PD-1 therapy. This could be related to the highest terminal half-life of tislelizumab compared to other ICB (23). Therefore, reducing the dose of tislelizumab or extending the interval of administration should be evaluated to improve safety in future studies on patients with post-allo-HSCT.

Some other factors may also cause the occurrence of GVHD after ICB therapy in posttransplantation patients. Although it is still controversial (22), two studies observed that a shorter interval between the transplantation and the first nivolumab infusion was associated with a higher risk of developing GVHD (15, 36). Extreme caution should be followed during the enrollment of patients with active cGVHD (21). Furthermore, the question remains as to whether anti-PD-L1 is safer than anti-PD-1. Hematopoietic cells upregulate the expression of both PD-L2 and PD-L1 after HSCT, but only PD-L1 is broadly expressed by parenchymal cells in host GVHD target tissues (47). Host PD-L1 is dominant over PD-L2 in regulating GVHD lethality (47), and the PD-L1 expression on donor T cells may drive GVHD lethality (53). Thus, PD-L1 may play a vital role in the development of GVHD. At present, there is still a lack of reliable data on the clinical application of anti-PD-L1 for posttransplantation patients, and the safety and efficacy of PD-L1 inhibitors need to be investigated clinically.

## Conclusion

Azacitidine in combination with anti-PD-1 seems to be a rational strategy for posttransplantation relapsed AML but needs further urgent clinical investigation. The report highlights the safety issues of an anti-PD-1 antibody in combination with azacitidine after allo-HSCT, especially GVHD. Additionally, we conducted an in-depth discussion around safety issues and provided suggestions for follow-up research. For such patients, the type, dosage, and timing of ICB drugs should be selected with caution.

## Data Availability Statement

The raw data supporting the conclusions of this article will be made available by the authors, without undue reservation.

## Ethics Statement

Ethical review and approval was not required for the study on human participants in accordance with the local legislation and institutional requirements. The patients/participants provided their written informed consent to participate in this study.

## Author Contributions

HL was involved in the identification, selection, and management of the patient and manuscript review. SY was involved in the management of the patient and manuscript drafting. CJ and QZ were involved in the selection and management of the patient and manuscript review. LY, ZB, HJ, NH, and ZB were involved in manuscript editing. HG was involved in the detection of samples. All authors have read and approved the final manuscript.

## Conflict of Interest

The authors declare that the research was conducted in the absence of any commercial or financial relationships that could be construed as a potential conflict of interest.
