# Supplementary material for: Case Report: Combination Therapy With PD-1 Blockade for Acute Myeloid Leukemia After Allogeneic Hematopoietic Stem Cell Transplantation Resulted in Fatal GVHD
Source: Front Immunol. 2021 Apr 1;12:639217. doi: 10.3389/fimmu.2021.639217 (PMC8047076; doi:10.3389/fimmu.2021.639217)
Supplement: Supplementary file 1 [file Table_1.DOCX]

Supplementary Table. The basic characteristics of the patient in diagnosis with t-MDS

| Morphological Examination | 9% blasts in BM, and 11% blasts in PB |
| --- | --- |
| Immunophenotyping by FC (BM) | 1.75% cells are positive for CD34, CD117, CD33, CD13, CD11c, CD38, partial positive for CD56, negative for HLA-DR, CD96, CD11b, CD16, CD42a, CD36, CD14, CD64, CD5, CD7, CD4, CD8, CD19, ckappa, clambda, CD20, kappa, lambda, CD10 in BM. |
| Karyotype (BM) | 46, XY, i(17), (q10)[16]/47, XY, +21[1]/46, XY[4].(1) |
| Tumor mutation detection by NGS | ETV6 mutation, SRTBP1 mutation |
| Immunohistochemical Staining | MPO (partial positive), CD235 (partial positive), Ki-67 (index about 60%), CD3 (scattered +), CD20 (-), CyclinD1 (-), CD10 (scattered +), PAX-5 (-), Bcl-2 (-), BCL (-), FVIII-R-Ag (macrophage+), CD (20-), Kappa (several+), namda (several+), CD79a-, CD23-. |

BM, bone marrow; PB, peripheral blood; FC, flow cytometry; NGS, next generation sequencing
